# Protein Copy Number Distributions for a Self-Regulating Gene in the Presence of Decoy Binding Sites

**DOI:** 10.1371/journal.pone.0120555

**Published:** 2015-03-26

**Authors:** Pavol Bokes, Abhyudai Singh

**Affiliations:** 1 Department of Applied Mathematics and Statistics, Comenius University, Bratislava, Slovakia; 2 Department of Electrical and Computer Engineering, University of Delaware, Newark, Delaware, USA; Technische Universität Dresden, Medical Faculty, GERMANY

## Abstract

A single transcription factor may interact with a multitude of targets on the genome, some of which are at gene promoters, others being part of DNA repeat elements. Being sequestered at binding sites, protein molecules can be prevented from partaking in other pathways, specifically, from regulating the expression of the very gene that encodes them. Acting as decoys at the expense of the autoregulatory loop, the binding sites can have a profound impact on protein abundance—on its mean as well as on its cell-to-cell variability. In order to quantify this impact, we study in this paper a mathematical model for pulsatile expression of a transcription factor that autoregulates its expression and interacts with decoys. We determine the exact stationary distribution for protein abundance at the single-cell level, showing that in the case of non-cooperative positive autoregulation, the distribution can be bimodal, possessing a basal expression mode and a distinct, up-regulated, mode. Bimodal protein distributions are more feasible if the rate of degradation is the same irrespective of whether protein is bound or not. Contrastingly, the presence of decoy binding sites which protect the protein from degradation reduces the availability of the bimodal scenario.

## Introduction

Gene expression has been characterised as pulsatile, with short bursts of intensive protein synthesis being interspersed by longer periods of quiescence [1–3]. As a consequence of bursting, cell-to-cell variability in protein abundance is much larger than would be expected, had proteins been produced one molecule at a time [4, 5]. In order to understand the implications of this variability, it is instrumental to examine the interplay between bursting dynamics and other biochemical pathways.

Pulsatile gene expression can be explained, conceptually as well as mathematically, by the random telegraph model [6], according to which a gene can transition between an inactive state and an active state, whereby transcription can be initiated only from the latter. Bursts occur if the active state is unstable but leads to rapid transcription [7].

The burst size, i.e. the number of products (here proteins) synthesised per burst, depends on the length of time spent in the active state. Provided that the inactivation mechanism consist of a single memoryless step, during which the synthesis rate is not subject to change, the size of a burst is geometrically distributed [8], with the mean given by the ratio of the production rate and the inactivation rate constants. If the mean burst size is large, the geometric distribution can be approximated by its continuous counterpart, the exponential distribution [9].

A mathematical model for gene expression dynamics in which production occurs spontaneously in bursts of exponentially distributed size, and is balanced by deterministic decay, predicts that the cell-to-cell distribution of protein level follows the gamma law [10]; the prediction is consistent with experimental results obtained for some model organisms [9].

Self-regulation (or auto-regulation), whereby a protein expressed from a gene activates its own transcription, is ubiquitous in gene networks [11]. The presence of a positive feedback confers two distinct scenarios on the expression of a gene: one in which the feedback loop is turned off and the gene is expressed at low levels, and another in which the feedback loop is established and the gene is expressed at relatively higher levels. The existence of two distinct, stable, modes of gene expression implies that a mechanism for preserving information on the cell’s history is available, which is of great importance in the context of cell-fate selection [12, 13].

The interaction between transcription factors and the DNA is not limited to self-regulation: key transcription factors bind to a multitude of target genes [14], as well as to other non-coding regions of the DNA [15]. An information-theoretical analysis has suggested that eukaryotic transcription factors engage in widespread non-functional binding [16]. Additional binding sites, functional or otherwise, sequester protein molecules at the expense of their engagement in the autoregulation loop, which is why we shall refer to them as decoy binding sites.

The HIV-1 Trans-Activator of Transcription (Tat) is an example of a transcription factor that regulates itself and binds to a large number of targets; more than half of these reside in the non-coding DNA’s repeat elements [17]. A synthetically engineered system in budding yeast which incorporates both self-regulation and decoy binding sites can serve as another example of a self-regulating gene which interacts with decoys [15].

The expression dynamics of a self-regulating gene in the presence of decoy binding sites has previously been modelled deterministically with differential equations [18], stochastically with a chemical master equation [18], and using a chemical Langevin equation [19]. The full master equation involves multiple reaction species, namely the promoter state, the bound protein and the free protein, which are interlinked in a nonlinear reaction network. Since analytic solution to such a high-dimensional problem is unavailable, one has to resort to stochastic simulation, numerical solution [20], or a physically justified dimensional reduction of the problem, in order to obtain a mathematical insight into the problem. While analytic results can be obtained from a Langevin approximation to the master equation, this approximation requires that the number of molecules change continuously in time [21], thus precluding the possibility of burst-like synthesis.

In this paper, a suitable reduction of dimension is achieved by adapting the framework of [10] to describe gene-expression bursting and by using a quasi steady-state approximation for the interaction between protein molecules and decoy binding sites. The latter methodology is introduced in the next section, and is used to describe the relationship between the amount of free protein and the total protein level (both free and bound), and to determine the rate of protein degradation as a function of either of these quantities. Having determined the decay rate, we then proceed to specify the mechanics of protein bursting and formulate, and solve, the master equation associated with the reduced gene-expression model. These results allow us to delineate the parametric regions in which the steady-state solution to the master equation is unimodal and in which it possesses two modes. The connection between the stochastic model and its deterministic counterpart is also analysed, and the implications of the possibility of having differential decay rates for free and bound proteins are investigated. Lastly, we summarise our modelling assumptions and the rationale for the chosen framework, comparing it to previous approaches, and provide an overview of new results.

## Analysis

### Reduction of dimension

In the full model for gene expression in the presence of decoy binding sites includes, one keeps track of (at least) two separate reaction species: the free protein and the bound protein. Dimensional reduction can be achieved if we assume that the interaction between the two, i.e. the binding and the unbinding, is so fast as to be at any time at a quasi steady state. The concentrations (the unit of concentration is arbitrary and can be set to 1 protein molecule per cell) *x*_f_ of free protein, *y*_f_ of free decoy binding sites, and *x*_b_ of the protein-binding site complex then satisfy

$$\begin{array}{c} x_{f}y_{f}=k_{b}x_{b}, \end{array}$$ (1)

where *k*_b_ is the dissociation constant for the interaction (the ratio of dissociation and association rate constants).

The total concentration *x* of protein, both bound and free, and the total decoy binding site concentration *y* are given by

$$\begin{array}{c} x=x_{f}+x_{b},\;y=y_{f}+x_{b}. \end{array}$$ (2)

The total binding site concentration does not change over time, i.e. *y* is a parameter. The other quantities will change in time, due to production and decay mechanisms described later.

Multiplying the second equation of (2) by *x*_f_ and then using (1), we obtain for the bound protein level a relationship

$$\begin{array}{c} yx_{f}=x_{b}\left(k_{b}+x_{f}\right)\;\Rightarrow \;x_{b}=\frac{yx_{f}}{x_{f}+k_{b}}, \end{array}$$ (3)

allowing us to determine the concentration of bound protein from that of free protein. The total protein concentration *x* comprises both free and bound molecules,

$$\begin{array}{c} x=x_{f}+\frac{yx_{f}}{x_{f}+k_{b}}. \end{array}$$ (4)

The inverse relationship to (4),

$$\begin{array}{c} x_{f}=\frac{x-y-k_{b}+\sqrt{{\left(k_{b}+y-x\right)}^{2}+4k_{b}x}}{2}, \end{array}$$ (5)

can be used to determine the concentration of free protein if the total protein concentration is given. Formulae (4) and (5) represent our dimensional reduction: it suffices to know the amount of free protein to determine the total protein level; conversely, the total protein level determines the proportions of bound and free molecules.

The rate of protein degradation *c* = *c*(*x*) is given by the sum of the degradation rate of free protein and that of bound protein,

$$\begin{array}{c} c\left(x\right)=\gamma_{f}x_{f}+\gamma_{b}\left(x-x_{f}\right), \end{array}$$ (6)

in which *γ*_f_ and *γ*_b_ are the degradation rate constants for the free and bound protein, and *x*_f_ depends on *x* as determined in (5).

We shall assume that *γ*_f_ = 1, which can be achieved by measuring time in the units of the lifetime of free protein. With this specification, the parameter *γ*_b_ is interpreted as the ratio between degradation rate constants for bound and free protein; hence, *γ*_b_ = 1 as long as the interaction with decoy binding sites does not affect the propensity of protein to be degraded; *γ*_b_ = 1 will also hold if proteins are stable and the decline in their concentration is due to cell growth. If *γ*_b_ < 1 (*γ*_b_ > 1), binding decoy sites diminishes (enhances) the propensity of protein molecules for degradation.

### The master equation

Proteins are transcribed in randomly-timed bursts, each burst leading to the synthesis of an exponentially distributed amount of protein, with the average burst size given by a parameter *b* [3]. The overall protein dynamics will be hybrid, with deterministic decay occurring with rate (6) being balanced by the stochastic burst-like production.

The probability density function *p*(*x*, *t*) of observing the protein at concentration *x* at time *t* satisfies the master equation [10, 22, 23]

$$\begin{array}{ccc} & & \frac{\partial p(x,t)}{\partial t}-\frac{\partial }{\partial x}\left(c\left(x\right)p\left(x,t\right)\right)\\ & & =\;\int_{0}^{x}\left(b^{-1}e^{-(x-x^{\prime })/b}-\delta \left(x-x^{\prime }\right)\right)a\left(x^{\prime }\right)p\left(x^{\prime },t\right)dx^{\prime }, \end{array}$$ (7)

where *a*(*x*) is the stochastic transcription rate, which may depend on the present level *x* of protein, owing to transcriptional autoregulation of the protein. The functional form of *a*(*x*) can be deduced from the details of molecular interaction between the protein and its gene.

We focus on the case of non-cooperative positive autoregulation, which is appropriate for the HIV-1 regulatory protein Tat [24, 25] as well as for the experimental system involving tTA driven by 1×tetO promoter [15], assuming the form of

$$\begin{array}{c} a\left(x\right)=a_{0}+\frac{a_{1}x_{f}}{k_{p}+x_{f}}, \end{array}$$ (8)

in which *a*_0_ is the basal transcription rate (per protein lifetime), *a*_1_ gives the difference between the up-regulated and the basal rates, and *k*_p_ is the dissociation constant for the interaction between the protein and its binding site at the promoter. The concentration *x*_f_ of free protein is obtained as a function of the total protein concentration *x* according to the expression (5).

Any decrease in free protein concentration resulting from the interaction between the protein and the functional binding sites is neglected because (eukaryotic) transcription factors are typically present at hundreds or thousands of copies, while a typical gene would be present at 1–2 copies and be regulated by a handful of functional sites. This would hold in the tTA system [15], where a single copy of a gene encoding the tTA protein is driven by a promoter containing a single TetO binding site (for the tTA protein), and would also extend to the HIV-1 regulatory protein Tat in the case of an infection by a single copy (or a small number) of the virus.

The stationary solution *p*(*x*, *t*) = *p*(*x*) to (7) can be shown to satisfy a simpler equation (see S1 Supporting Information),

$$\begin{array}{c} \frac{d}{dx}\left(c\left(x\right)p\left(x\right)\right)+\frac{c(x)p(x)}{b}=a\left(x\right)p\left(x\right), \end{array}$$ (9)

from which, by direct integration,

$$\begin{array}{c} p\left(x\right)=\frac{\kappa }{c(x)}\exp\left(-\frac{x}{b}+\int \frac{a(x)}{c(x)}dx\right), \end{array}$$ (10)

where *κ* is a normalisation constant; such a form has been disclosed in previous studies [10, 22].

For the specific choices of the burst rate (8) and decay rate (6), Equation (10) reduces to a closed expression (see S1 Supporting Information for details)

$$\begin{array}{ccc} p(x) & = & \kappa \;\exp\left(-\frac{x}{b}\right)x_{f}^{q_{1}-1}{\left(x_{f}+k_{p}\right)}^{q_{2}}\\ & \times & {\left(x_{f}+k_{b}\right)}^{q_{3}+1}{\left(x_{f}+k_{b}+\gamma_{b}y\right)}^{q_{4}-1}, \end{array}$$ (11)

where

$$\begin{array}{cc} q_{1} & =a_{0}\frac{k_{b}+y}{k_{b}+\gamma_{b}y}, \end{array}$$ (12)

$$\begin{array}{cc} q_{2} & =\frac{a_{1}\left(k_{p}-k_{b}\right)}{k_{p}-k_{b}-\gamma_{b}y}\left(1+\frac{yk_{b}}{{\left(k_{p}-k_{b}\right)}^{2}}\right), \end{array}$$ (13)

$$\begin{array}{cc} q_{3} & =\frac{1}{\gamma_{b}}\left(\frac{a_{1}k_{b}}{k_{p}-k_{b}}-a_{0}\right), \end{array}$$ (14)

$$\begin{array}{cc} q_{4} & =\left(\gamma_{b}y+\frac{k_{b}}{\gamma_{b}}\right)\left(\frac{a_{0}}{k_{b}+\gamma_{b}y}+\frac{a_{1}}{k_{b}-k_{p}+\gamma_{b}y}\right). \end{array}$$ (15)

The free protein level *x*_f_ is understood in (11) to be a function of *x*, as given by expression (5). The formula (11) for the stationary distribution is valid as long as some special cases, namely that of *k*_p_ = *k*_b_, or *k*_p_ = *k*_b_+*γ*_*b*_ *y*, or *γ*_*b*_ *y* = 0, are avoided; results for these special cases are given in S1 Supporting Information.

One can argue that the activity of a gene is determined by the concentration *x*_f_ of free proteins, rather than the total protein concentration *x*. Therefore, it is of interest to express the above results in terms of the probability density function $\tilde{p}(x_{\text{f}})$ of *x*_f_. The transformation rule implies that

$$\begin{array}{ccc} \tilde{p}\left(x_{f}\right) & = & p\left(x\left(x_{f}\right)\right)\frac{dx}{dx_{f}}\\ & = & p\left(x\left(x_{f}\right)\right)\left(1+\frac{yk_{b}}{{\left(x_{f}+k_{b}\right)}^{2}}\right), \end{array}$$ (16)

in which *p*(*x*(*x*_f_)) can be evaluated using the expressions (11), in which, this time round, the free protein concentration *x*_f_ takes the role of the independent variable while *x* is understood to be a function of *x*_f_, as given by (4).

Should one prefer to measure the protein concentration on a logarithmic scale, i.e. using *z* = log_10_(*x*), the probability density function $\overset{˘}{p}(z)$ of *z* is given by

$$\begin{array}{c} \overset{˘}{p}\left(z\right)=p\left(10^{z}\right)\frac{d(10^{z})}{dz}=p\left(10^{z}\right)10^{z}\ln\left(10\right), \end{array}$$ (17)

which is defined for any value of *z*. To measure the free protein concentration on the logarithmic scale, the transformation should be applied to $\tilde{p}(x_{\text{f}})$ instead of *p*(*x*).

**Fig 1:**
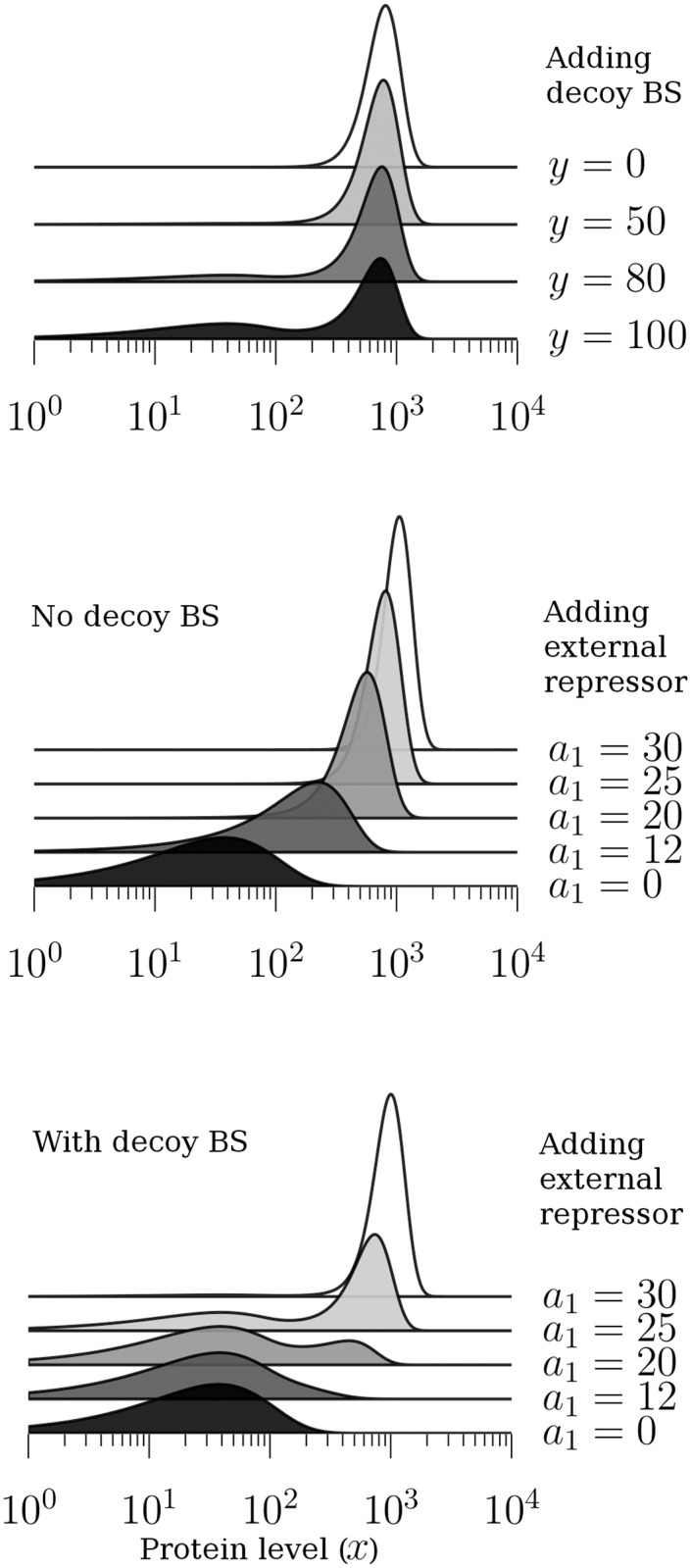
Protein level distributions with and without decoy binding sites. *Top:* Probability density function of the total protein concentration on a logarithmic scale. The number *y* of binding sites is varied between 0 and 100. The decay rate constants *γ*_f_ and *γ*_b_ for free and bound protein are both set to one; the dissociation constant *k*_b_ for the interaction with binding sites is set to one molecule; the dissociation constant *k*_p_ for the interaction with the promoter is set to 500 molecules; the basal transcription rate is 0.75 bursts per protein lifetime and the regulable transcription rate is 25 bursts per lifetime. The mean burst size is 50 molecules. *Middle and bottom:* The gene-expression response to changes in *a*_1_ is graded in the absence of decoy binding sites (*y* = 0, *middle*); it is binary if decoy binding sites are present (*y* = 100, *bottom*). The other model parameters are chosen as follows: *γ*_f_ = *γ*_b_ = 1, *k*_b_ = 1, *k*_p_ = 500, *a*_0_ = 0.75, *b* = 50.

In Fig. 1, top, we show the probability density function of the total protein concentration on a logarithmic scale for changing levels of decoy binding site concentration (0, 50, 80 and 120 sites per cell). Following previous studies [15], the rate of decay for protein molecules that are bound to decoy binding sites is set to be equal to the decay rate for free protein (*γ*_b_ = 1), and the protein is assumed to bind to decoy sites much more tightly than to its promoter (*k*_b_ = 1 ≪ *k*_p_ = 500, cf. [15]). The transcription rate is set to start at a low basal value of *a*_0_ = 0.75 bursts per protein lifetime and can be increased by *a*_1_ = 25 bursts per lifetime in case of full promoter occupancy, and the mean burst size is set to *b* = 50. Such choices ensure that at the basal rate of expression, a large fraction of protein molecules is sequestered away by decoy binding sites; on the other hand, the relatively high regulable rate ensures that the positive feedback loop is established once there are enough protein copies. Indeed, the figure suggests that adding decoy binding sites leads to a bimodal distribution of protein level, the lower mode corresponding to the basal expression scenario and the higher mode corresponding to the upregulated scenario.

Fig. 1, middle and bottom, illustrates the response of protein distribution to changes in the dose of an external repressor in the absence (Fig. 1, middle) or in the presence (Fig. 1, bottom) of decoy binding sites. For simplicity, we assume that the effect of the repressor is in reducing the efficiency of the autoregulation loop, i.e. reducing *a*_1_. Such an effect is easy to model with the current mathematical model; at the same time, it is equivalent to more complex repressing mechanisms, such as the protein-protein interaction between the dox repressor and tTA protein [15]. While in the absence of decoy binding sites the response of the protein distribution is graded (Fig. 1, middle), in their presence the response is binary (Fig. 1, bottom).

### Exploring the parameter space

As has been illustrated on specific examples in the previous section, our stochastic model allows for a bimodal distribution of protein level, especially if decoy binding sites are present. In this section we seek to delineate the regions of the parameter space for which bimodality, as opposed to unimodality, is observed. In doing so we follow a strategy that was previously used in [26] to explore the parameter space of a stochastic gene expression model in the absence of decoy binding site. We extend this strategy by considering bimodality not only on linear, but also on logarithmic scale.

Bimodality of protein distribution can occur only if there are multiple local extrema of *p*(*x*), i.e. multiple solutions to the equation d*p*(*x*)/d*x* = 0. Differentiating the product on the left-hand side of (9) and regrouping the terms yields

$$\begin{array}{c} c\frac{dp}{dx}=\left(a-\frac{c}{b}-\frac{dc}{dx}\right)p, \end{array}$$ (18)

implying that for local extrema the bracketed term on the right-hand side of (18) must vanish.

For simplicity, we shall assume that *γ*_b_ = 1, i.e. that protein molecules decay with the same rate constant whether they are free or bound to decoy sites. The general case of unequal decay poses challenges that are technical, but not fundamental in their nature, and can be treated by the same methods as used below for *γ*_b_ = 1.

We recall that the gene response function *a* and of the decay rate *c* satisfy

$$\begin{array}{ccc} a & = & a_{0}+\frac{a_{1}x_{f}}{k_{p}+x_{f}},\\ c & = & x=x_{f}+\frac{yx_{f}}{x_{f}+k_{b}},\;\frac{dc}{dx}=1. \end{array}$$ (19)

Therefore, the derivative d*p*/d*x* is equal to zero at a given total protein level *x* if for the associated free protein level *x*_f_ we have

$$\begin{array}{c} a_{0}+\frac{a_{1}x_{f}}{k_{p}+x_{f}}-\frac{x_{f}}{b}-\frac{yx_{f}}{b(x_{f}+k_{b})}-1=0. \end{array}$$ (20)

The number of local extrema changes if the right-hand side of (20), as a function of *x*_f_, crosses the value zero tangentially, i.e. if

$$\begin{array}{c} \frac{a_{1}k_{p}}{{\left(k_{p}+x_{f}\right)}^{2}}-\frac{1}{b}-\frac{yk_{b}}{b{\left(x_{f}+k_{b}\right)}^{2}}=0 \end{array}$$ (21)

holds in addition to (20). For any fixed level of *x*_f_, Equations (20) and (21) specify the combinations of parameter values (*a*_0_, *a*_1_, *k*_p_, *b*, *y*, and *k*_b_) for which a transition from unimodality to bimodality in *p*(*x*) occurs at *x* corresponding to the fixed value of *x*_f_. As the value of *x*_f_ is varied, the parameter values satisfying (20) and (21) span the hyper-surface in the parameter space that separates the region in which bimodality is observed from that in which unimodality occurs.

Instead of grappling with the 6-dimensional geometry (5-dimensional after a suitable scaling) of the parameter space, it is fruitful to try to understand one of its 2-dimensional cross-sections, obtained by prescribing the values for all but two parameters. Hence, if we fix the values of the regulable transcription rate *a*_1_, mean burst size *b*, decoy binding site concentration *y* and the dissociation constant *k*_b_ for the binding to decoy sites, we can use (20) and (21) to determine the curves that delineate in the (*a*_0_, *k*_p_)-cross-section of the parameter space the regions of bimodality and unimodality.

Before doing that, we need to clarify an important point about bimodality itself. If a random variable has a distribution which is bimodal, the distribution of a non-linear transformation of the variable, even if it be a monotone one, need not be bimodal (and vice versa). As gene-expression measurements are often reported on a logarithmic scale, it is of interest to examine the conditions for bimodality of the probability density function $\overset{˘}{p}(z)$ of the log-scaled total protein concentration *z* = log_10_(*x*).

The relationship (17) between the linear-scale and logarithmic-scale densities *p*(*x*) and $\overset{˘}{p}(z)$ can be expressed in terms of the linear variable *x* as

$$\begin{array}{c} \overset{˘}{p}\left(\log_{10}\left(x\right)\right)=p\left(x\right)x\text{ln}\left(10\right), \end{array}$$ (22)

implying that

$$\begin{array}{ccc} \frac{d\overset{˘}{p}}{dx} & = & \ln\left(10\right)\left(x\;\frac{dp}{dx}+p\right)\\ & = & \frac{\ln(10)\;p\;x}{c}\left(a-\frac{c}{b}-\frac{dc}{dx}+\frac{c}{x}\right), \end{array}$$ (23)

in which we used (18) to express the derivative d*p*/d*x*.

Assuming, as before, that free and bound protein decay with the same rate constant, we have *c* = *x*, and the last two terms of the bracketed expression in (23) cancel each other; therefore the derivative $\text{d}\overset{˘}{p}/\text{d}x$ vanishes at a given protein level *x*, and so does the derivative $\text{d}\overset{˘}{p}/\text{d}z$ at the associated log-scaled level *z* = log_10_(*x*), if

$$\begin{array}{c} a_{0}+\frac{a_{1}x_{f}}{k_{p}+x_{f}}-\frac{x_{f}}{b}-\frac{yx_{f}}{b(x_{f}+k_{b})}=0 \end{array}$$ (24)

holds for the free protein concentration *x*_f_. The number of local extrema of $\overset{˘}{p}$ changes if the left-hand side of (24) crosses zero tangentially, i.e. if, in addition to (24), the derivative of the left-hand side of (24) with respect to *x*_f_ vanishes. Since the left-hand side of (24) differs only by an additive constant from that of (20), the condition of tangential crossing is given by (21).

Equations (24) and (21), in which *x*_f_ is allowed to vary arbitrarily, specify a hyper-surface in the parameter space which encloses the region of bimodality of the logarithmic-scale density. Since any solution to (20)–(21) can be turned by means of the translation *a*_0_ → *a*_0_−1 into a solution to (24)–(21), the hyper-surface is a translation, by 1 to the left along the *a*_0_ axis, of the hyper-surface enclosing the region of bimodality of the linear-scale density.

**Fig 2:**
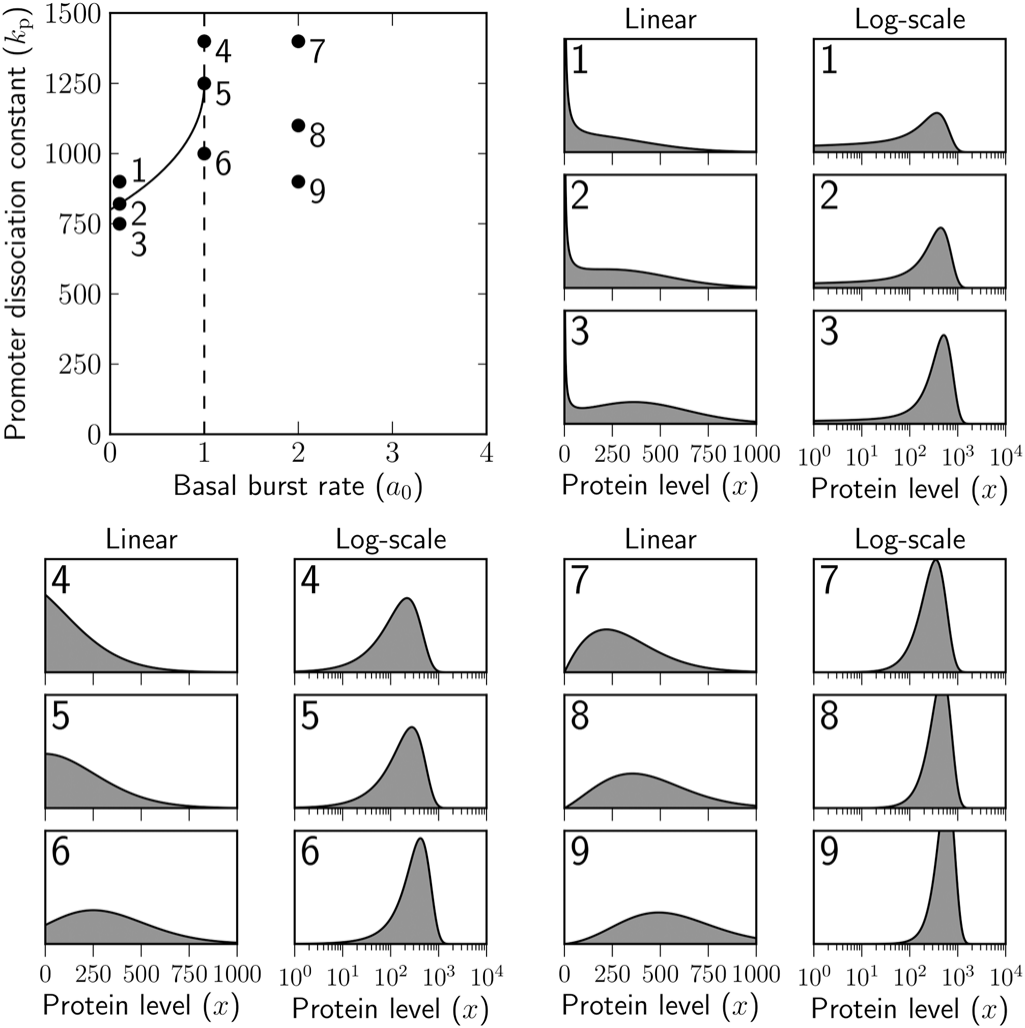
Bimodality and unimodality for the linear-scale and logarithmic-scale densities of total protein concentration in the absence of decoy binding sites. The basal transcription rate *a*_0_ (per protein lifetime) and the dissociation constant for promoter binding *k*_p_ (measured in the units of molecule number) are varied, the others being prescribed fixed values, namely the regulable transcription rate *a*_1_ is set to 25 bursts per protein lifetime, the number *y* of decoy binding sites is set to zero and the mean burst size *b* is equal to 50 molecules.

**Fig 3:**
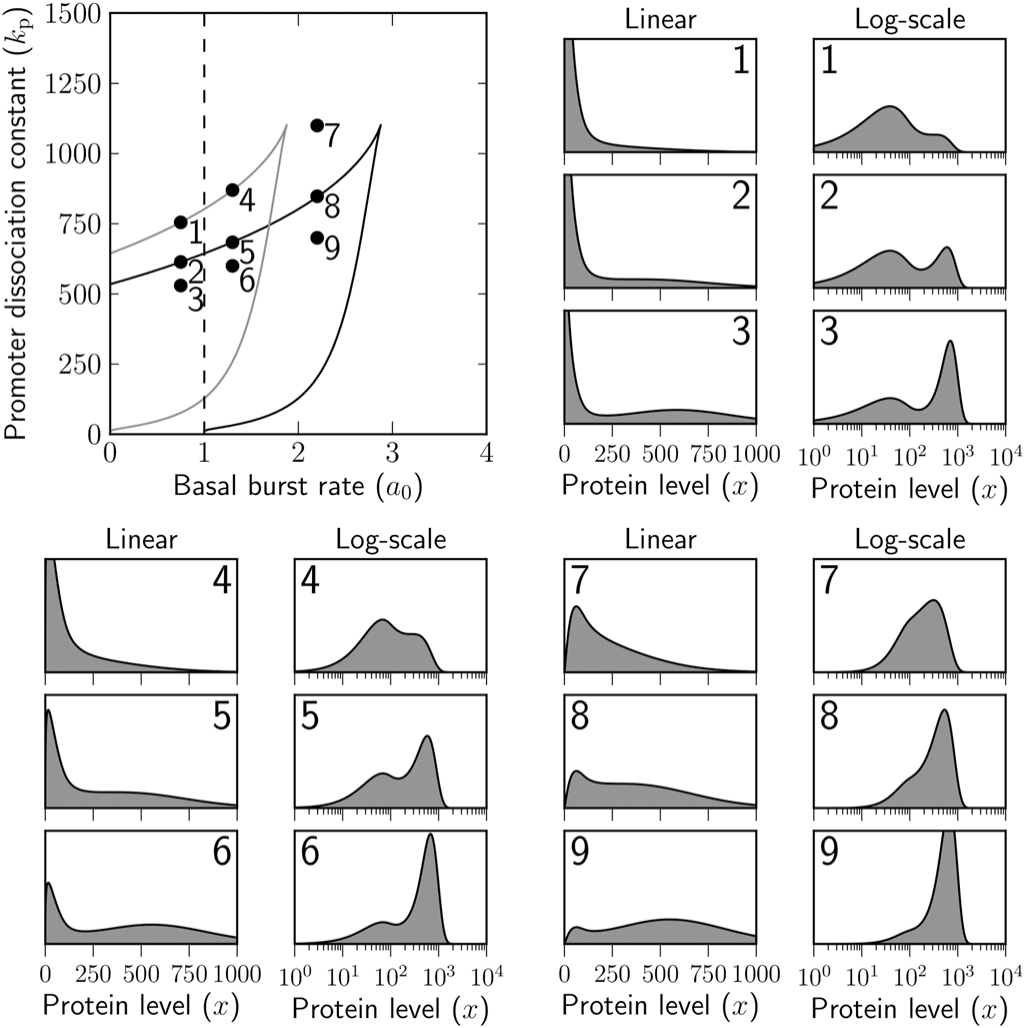
Bimodality and unimodality for the linear-scale and logarithmic-scale densities of total protein concentration in the presence of decoy binding sites. The basal transcription rate *a*_0_ (the average number of basal bursts per protein lifetime) and the dissociation constant for promoter binding *k*_p_ (number of molecules required for promoter half-occupancy) are varied, the others being prescribed fixed values: the regulable transcription rate *a*_1_ is set to 25 bursts per protein lifetime, there are *y* = 100 decoy binding sites to which the proteins can bind at dissociation constant *k*_b_ = 1 molecule; the burst size has the mean of *b* = 50 molecules and the proteins are degraded with unit rate whether they are bound or not, i.e. *γ*_f_ = *γ*_b_ = 1.

In Figs. 2 and 3 we show two cross-sections of the parameter space obtained by fixing the regulable transcription rate *a*_1_ = 25, the dissociation constant for decoy site binding *k*_b_ = 1, the mean burst size *b* = 50, and the number of decoy binding sites to either *y* = 0 (Fig. 2) or *y* = 100 (Fig. 3). The two remaining model parameters, the basal transcription rate *a*_0_ and the dissociation constant for promoter binding *k*_p_ are varied over realistic ranges 0 < *a*_0_ < 4 and 0 < *k*_p_ < 1500. Nine parameter sets from either cross-section are selected for which the linear-scale and logarithmic densities are shown. Alternative versions of Fig. 3 obtained by choosing different parameter values can be found in S1 Fig.

A rough picture of the cross-sections can be fleshed out by referring back to the physical interpretation of our model. If the basal rate *a*_0_ is low, any time the protein concentration decreases by chance to levels that are significantly lower than the dissociation constant for promoter binding, its autoregulation loop will be switched off for long periods of time, so that there would be a high probability of finding a low number of protein copies in the cell. Restarting the autoregulation loop will be more difficult in the presence of decoy binding sites, which will sequester the proteins produced in the first few bursts of basal expression. Higher levels of basal rate will make it easier for the protein levels to escape this trap, and with high probability a large number of proteins will be present in the cell.

On the other hand, if the dissociation constant for promoter binding is very low, it will require a relatively small amount, perhaps as small as produced in a few average-sized bursts, of protein molecules to turn on the autoregulation loop. A low value of the dissociation constant will therefore have a similar effect like a high value of the basal rate. If the dissociation constant is very large, perhaps as large as the maximum production rate (*a*_0_+*a*_1_)×*b*, it will be almost impossible for the protein to turn its production on, thus becoming trapped in the low-expression state for good.

While a sound physical understanding of the model can elucidate the basic logic of the parameter space, mathematical analysis needs to be put to use in order to paint a finer picture of it, which is interesting especially for the intermediate values of the basal rate *a*_0_ and the dissociation constant *k*_p_.

In the absence of binding sites, cf. Fig. 2, the response of the linear-scale density to an increase in promoter affinity, i.e. to a decrease in the dissociation constant *k*_p_, is binary if the basal rate *a*_0_ is very low (parameter sets 1–3 in Fig. 2). For high values of *k*_p_, much of the probability mass is concentrated at the low-expression singular peak (parameter set 1). As *k*_p_ decreases down to a critical level, indicated by the solid black curve in the figure, the linear-scale density acquires an inflection point (cf. parameter set 2), which then develops into a regular peak of high expression with further improvement of promoter affinity (parameter set 3). We note that unless *a*_0_ is very low and *k*_p_ is close to the critical level, the low-expression model will contain only a very small fraction of the entire probability mass (*a*_0_ = 0.1 for parameter set 3), with the rest of it residing around the regular high-expression peak.

The solid black curve of Fig. 2, on which the transition from unimodality to bimodality occurs, has been determined by solving the system of algebraic Equations (20)–(21) numerically in unknowns *a*_0_ and *k*_p_ for a range of values of *x*_f_. The curve encloses the region of bimodality from above; the dashed line *a*_0_ = 1 forms the border on the right-hand side. In order to understand the nature of the transition from bimodality to unimodality at *a*_0_ = 1, we note that if *a*_0_ ≥ 1, then the linear-scale density (11) is bounded from above, while it has a singularity at *x* = 0 if *a*_0_ < 1. As *a*_0_ converges to one from left, the singularity becomes ever thinner, until it peters out at *a*_0_ = 1 (see parameter set 6 of Fig. 2 as compared to parameter set 3), where the linear-scale density *p*(*x*) has a finite limit as *x* tends to zero; if *a*_0_ exceeds one, the density vanishes at *x* = 0 (see parameter sets 7, 8 or 9 of Fig. 2).

The logarithmic-scale density is always unimodal in the absence of binding sites, as is evidenced by the examples of Fig. 2; indeed, Equation (24) for local extrema of the logarithmic-scale density can easily be shown to possess one solution at most if *y* = 0. For low rates of basal transcription, the logarithmic density is highly skewed and possesses a fat left tail.

The parameter-space cross-section in which the number of decoy binding sites is set to *y* = 100 is visibly different, see Fig. 3, from the previous one. Binding sites are able to sequester the produce of basal transcription, thus stabilising the low-expression mode. The response of the linear-scale, as well as logarithmic scale, densities to an increase in binding affinity to the promoter (i.e. to decreasing *k*_p_) is binary for a relatively wide range of basal rates (see the parameter sets selected in Fig. 3). As *k*_p_ descends to a critical level, indicated by the upper branch of the solid black curve, the linear-scale density acquires an inflection point which turns into a peak of high expression upon further decrease in *k*_p_ (parameter sets 2, 5 and 8 of Fig. 3). On the lower branch of the solid black curve, it is the low-expression peak that turns into an inflection point (not shown; however, parameter set 9 of Fig. 3 is close to such a scenario).

The two branches of the solid black curve enclose the parameter region in which the linear-scale density is bimodal. The region of bimodality for the logarithmic-scale density is obtained, as explained above, by shifting these two branches by one unit to the left. The shifted curve is shown in grey; the parameter sets 1 and 4 that lie on the grey curve are seen to exhibit a transition, via an inflection point, between unimodality and bimodality on the logarithmic scale.

Thus, in comparison with the situation that arises in the absence of decoy binding sites, in their presence the probability mass is evenly distributed between the modes of low and high expression for a wide ranges of *a*_0_ and *k*_p_. Bimodality is observed not only on the linear scale, but also on the logarithmic one.

### Deterministic model

We multiply the master Equation (7) by *x* and integrate over all positive values of *x*, finding that the mean protein concentration satisfies

$$\begin{array}{c} \frac{d\langle x\rangle }{dt}=-\left\langle c\left(x\right)\right\rangle +b\left\langle a\left(x\right)\right\rangle . \end{array}$$ (25)

Equation (25) is not closed in the mean since the transcription and degradation rates are, in general, nonlinear functions of *x*.

Neglecting any fluctuations in (25), we obtain a deterministic formulation of the model,

$$\begin{array}{c} \frac{dx}{dt}=-c\left(x\right)+ba\left(x\right), \end{array}$$ (26)

which is an autonomous nonlinear first-order differential equation in protein concentration *x*.

At steady state, we have *ba*(*x*) = *c*(*x*), i.e.

$$\begin{array}{c} ba_{0}+\frac{ba_{1}x_{f}}{k_{p}+x_{f}}=x_{f}+\frac{\gamma_{b}yx_{f}}{x_{f}+k_{b}}, \end{array}$$ (27)

having expressed the transcription and degradation rates in terms of the free protein concentration, as given by Equations (6) and (8).

Equation (26) is bistable if there are multiple solutions *x*_f_ to (27). Notably, if there are no decoy binding sites (*y* = 0), or if proteins bound to decoy binding sites are not degraded (*γ*_b_ = 0), then (27) possesses a single solution, and bistability cannot occur.

In the linear degradation case, i.e. for *γ*_b_ = 1, the steady-state Equation (27) is equivalent to the Equation (24) for the extrema of the logarithmic-scale density of the stochastic model. Consequently, the deterministic model exhibits bistability if the stochastic model is bimodal on the logarithmic scale, i.e. for parameter values from the region which is enclosed by the grey curve in Fig. 3.

### Decoy sites and protein degradation

In regards to the impact of binding to decoy sites on protein stability, it is enlightening to juxtapose the following two contrasting cases, cf. [18]: (i) protein molecules are degraded with the same rate regardless of whether they are bound or not; (ii) they are protected from decay while bound. Having previously explored the former in detail, we now discuss the latter possibility; more details can be found in [23].

If bound proteins are stable, then sequestering of protein molecules at decoy binding sites decreases the effective protein degradation rate constant, thus increasing the mean steady-state protein level. Unlike in the previous case, adding decoy binding sites does not stabilise the low-expression mode: the effective increase in the autoregulation loop threshold, to which we attributed the stabilisation of the low-expression mode previously, will be balanced in its effect by the increase in the mean protein level.

**Fig 4:**
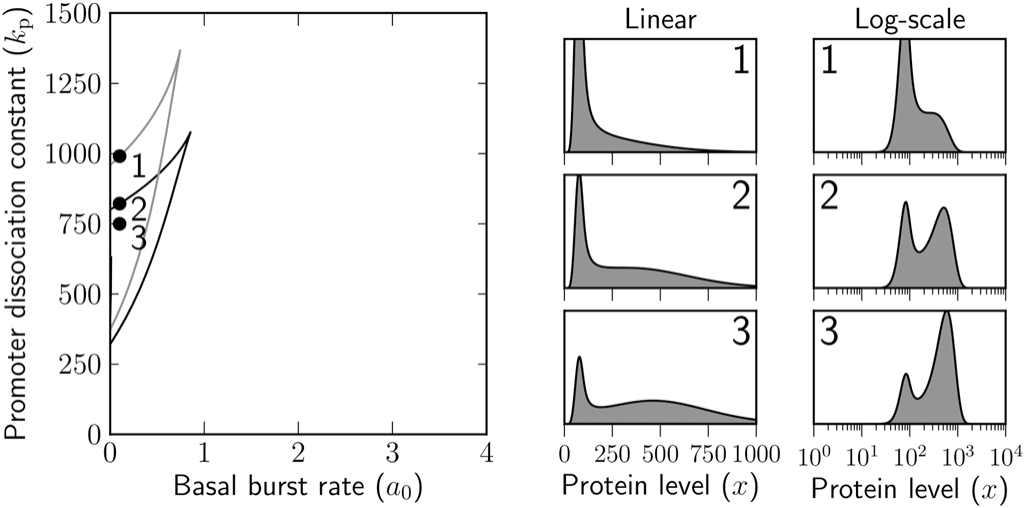
Bimodality and unimodality for the linear-scale and logarithmic-scale densities of total protein concentration in the presence of decoy binding sites, which protect the protein from degradation. The basal transcription rate *a*_0_ and the dissociation constant for promoter binding *k*_p_ are varied, the regulable transcription rate is set to *a*_1_ = 25 bursts per protein lifetime, the number of binding sites is *y* = 100 with which protein molecules interact at dissociation constant of *k*_b_ = 1 molecule; the mean burst size is *b* = 50; protein molecules are degraded with unit rate constant *γ*_f_ = 1 if they are free, but no degradation occurs if they are bound to decoys (*γ*_b_ = 0).

Fig. 4 shows the cross-section of the parameter space of our model in which the basal transcription rate *a*_0_ and the dissociation constant *k*_p_ for promoter binding are varied, while the remaining parameter values are fixed at constant values. The fixed values are the same as those used in Fig. 3, except for the degradation rate constant *γ*_b_ for bound protein, which is set to zero.

The critical values of *a*_0_ and *k*_p_, for which a transition between unimodality and bimodality in the linear-scale density occurs, form the black curve in Fig. 4. The grey curve separates the bimodal and unimodal regions of the logarithmic-scale density. The curves were determined using a modification of the method used to classify the parameter space in the case of *γ*_b_ = 1.

Comparing Figs. 2 and 4, we note that if *γ*_b_ = 0, then adding decoy binding sites does not lead to bimodal expression on the linear scale, unless bimodality is already observed in their absence. In the right panel of Fig. 4, we show the linear and logarithmic scale densities for *a*_0_ = 0.1 and three specific choices of *k*_p_. The densities have been calculated by taking the limit, as *γ*_b_ tends to zero, of (11). The response of the density, on either scale, to an increase in promoter binding affinity (i.e. to a decrease in *k*_p_) is binary. In contrast with the response observed at similarly low levels of *a*_0_ in the absence of binding sites (parameter sets 1–3 in Fig. 2), the lower peak is regular and can also be identified on the logarithmic scale.

Thus, adding decoy binding sites that protect the protein from degradation increases the mean protein expression level and decreases the heterogeneity in protein expression. On the other hand, if decoy binding sites are added that do not interfere with protein decay, protein expression decreases in the mean and can become more heterogeneous.

## Discussion

The copy number of protein molecules, expressed from a single gene in a population of isogenic cells, can vary dramatically from one cell to another. One of the key contributors toward this variability is the synthesis of gene products in bursts—spells of rapid production, which are interspersed by longer periods of gene inactivity.

Some proteins—also known as transcription factors—can bind to the DNA and regulate transcription; in particular, they may regulate the very gene that encodes them. A typical transcription factor would also bind to a host of other targets on the DNA. These targets are referred to as decoy binding sites, as they interfere with the autoregulatory loop by competing for free transcription factor molecules.

Our aim has been to investigate a minimal mathematical model which incorporates burst-like production of a protein, its self-regulation, and binding to decoy binding sites. In doing so, we made a number of simplifying assumptions, the justification of which is discussed next.

### Modelling assumptions

#### Fast mRNA turnover

In the presented model, new protein molecules are synthesised in bursts of random size, at a rate which is determined by the number of free proteins available for autoregulation. The model does not explicitly include the dynamics of mRNA intermediaries. Gene expression is a two-stage process [27], in which genes are first transcribed into mRNA molecules, which are subsequently translated into protein molecules or degraded. Transcription leads to bursts of mRNA synthesis, rather than protein synthesis; nevertheless, provided that the mRNA half-life is short, in comparison with that of the protein (as is often the case biologically), any mRNA burst will immediately be followed by a protein one [28]. Alternatively, should mRNAs be produced one molecule at a time—i.e. not in bursts —, protein bursts may still arise at the translation stage if, within its brief life span, an mRNA molecule is translated into a large number of proteins [29].

#### Exponential burst sizes

The burst size—the number of protein molecules in a burst—is proportional to the time that the gene spends in the active state, or, in case of translational bursts, to the mRNA life span. As have been demonstrated in literature [1, 9], these often follow the exponential distribution, to which we adhere in this paper.

#### The separation between concentration scales

Since burst-like protein production leads to large changes in molecule numbers, the contribution of protein production to the overall gene-expression noise dwarfs those of degradation and interaction with decoy sites, which involve only one protein molecule at a time. Therefore, we treat the latter processes as deterministic.

The separation between the typical concentration scale for the protein level and the molecular scale also allows us to neglect the necessary reduction in the amount of free protein due to its interaction with the autoregulatory binding sites. On the other hand, the reduction in the availability of free protein due their binding to decoy binding sites cannot be neglected: decoy binding sites may be present at levels comparable to those of the protein.

#### The separation between temporal scales

The final simplifying assumption that deserves special mention is that of a separation in timescales. As is common in mathematical modelling of gene regulation [30], we assume that the binding of protein molecules to the DNA equilibrates on a timescale which is appreciably faster than that of protein turnover. Performing a quasi-steady-state approximation [31], we equated the rates of binding and dissociation, thereby obtaining an explicit functional correspondence between the total protein amount and the number of free proteins at any time: as a result, our model is one-dimensional, tracking the dynamics of a single species (total protein concentration), which is being mirrored by the other species (free protein) according to their functional relationship (5).

### The modelling framework

The evolution in time, of the cell-to-cell distribution for the total protein level, is described by master Equation (7). It is an integro-differential equation, in which protein degradation is manifested by the convective term, while burst-like synthesis manifests itself in the integral term. The total degradation rate in the convective term is given by (6) as a sum of the degradation rate of free protein and that of bound protein (the rate constants for the two being not necessarily equal). The burst rate in the integral term increases with the number of free protein molecules, the functional dependence being of the Michaelis-Menten form (8), corresponding to non-cooperative positive autoregulation: examples of our interest, HIV-1 Tat protein and the synthetic yeast system, exhibit this kind of autoregulation [15, 24].

The exact stationary distribution (10) of the total protein level is found by solving, at steady state, master Equation (7), along the lines of [10]. The analytic solution is given by a closed-form expression, up to a normalisation constant which has to be determined by numerical integration. The free protein and logarithmic-scale (total or free protein) distributions can be obtained from the total protein distribution using the transformation rule.

### Connection with previous work

The cell-to-cell distribution of protein copy number for a self-regulating gene, which is being expressed in bursts, has been characterised in a number of previous studies using mathematical models based on the chemical master equation, to a varying degree of detail [10, 32]. In these models, however, decoy binding sites have not been included.

A fine-grained mathematical model for a self-regulating gene in the presence of decoy binding sites, accounting for individual promoter transitions, DNA binding and dissociation, etc., has been investigated in [18]. The master equation associated with the fine-grained model was solved numerically [18]. Some analytic results have been obtained using a Langevin approximation to the master equation [19].

Our paper extends the model of [10] by including the interaction between the protein and decoy binding sites. Our results also contribute, by finding an analytically tractable stationary protein distribution, to the previous approaches in modelling the dynamics of a self-regulating protein in the presence of decoys due to [18, 19] and [15].

Conditions for the protein copy number distribution to possess two modes are derived in a similar manner as was done in [26] for the model of [10] without decoy binding sites. In comparison to [26], bimodality is investigated not only on the linear, but also on the logarithmic scale. Our model also represents a biologically important example to which the results of [22] on the existence, uniqueness, and long-time behaviour of solutions to a master equation such as (7), are applicable.

### New results

Fig. 1 illustrates some basic properties of the stationary distribution (for the total protein number, on a logarithmic scale). Most parameter values are adopted from [15], in which a deterministic version of our model was studied and compared to experimental measurements on a synthetic yeast system. Specifically, the interaction between the transcription factor and decoy binding sites is assumed not to affect the propensity for the protein to be degraded; secondly, decoy binding sites are assumed to attract proteins with much greater affinity than the autoregulatory binding site.

Adding decoy binding sites is shown to lead to bimodality in the protein distribution (Fig. 1, top). Biologically, decoy binding sites sequester much of the produce of basal gene expression, at the expense of autoregulation, leading to the stabilisation of the basal expression mode, and to a bimodal profile of the distribution.

The middle and bottom panels of Fig. 1 illustrate the response of the distribution to changing doses of an upstream repressor (modelled, for simplicity, by varying the parameter corresponding to the maximal achievable transcription rate). In the absence of decoys (Fig. 1, middle), the response is graded, while in their presence, the response is binary (Fig. 1, bottom), a distinction which is in qualitative agreement with empirical distributions for a fluorescent reporter protein in the synthetic yeast system [15].

The tenacity of the basal expression mode is sensitive to changes in the basal transcription rate *a*_0_ and the affinity of the transcription factor for its autoregulatory site (this being equal to the reciprocal of the dissociation constant *k*_p_). As *a*_0_ and *k*_p_ vary, the total protein distribution can transition between unimodal and bimodal behaviour. In the absence of decoys (cf. Fig. 2), bimodality is observed on the linear scale only if basal transcription occurs at a rate lower than one burst per protein lifetime. If observed, the basal expression mode is always singular. On the logarithmic scale, the distribution is unimodal for all combinations of parameters, being highly skewed in the regions of parameter space where the linear-scale distribution is bimodal. In the presence of decoys, bimodality is observed for a broader region of parameter values, both on the linear and also on the logarithmic scale (cf. Fig. 3). On the linear scale, the low expression mode can be either singular or regular.

Bimodality of stationary distributions in stochastic models is often thought of as a counterpart of bistability in deterministic systems. We have shown that the conditions for bistability for a deterministic reduction of our model coincide with the conditions for bimodality on the logarithmic scale.

The above results have been concerned with the case of equal degradation for free and bound protein. A radically different scenario follows if decoy binding sites protect the protein from being degraded. If such decoys are added, bimodal behaviour is observed for a narrower region of parameter values than in their absence (cf. Fig. 4). The effect of adding decoy binding sites is one of an increase in the protein level, due to a reduction in the overall degradation rate, rather than a stabilisation of the low expression mode. In the presence of protective decoys, the basal expression mode becomes regular and is maintained on the logarithmic scale.

If free protein molecules are degraded with a different rate constant than the bound ones, conditions for bimodality no longer coincide with those for bistability of the deterministic model; indeed, if bound proteins are completely protected from degradation, bistability cannot occur, while bimodality on the logarithmic scale can.

In summary, we propose a minimalistic model for burst-like gene expression for a transcription factor that can regulate itself as well as bind to a large number of targets on the DNA. Our main results are the derivation of an exact stationary distribution for the protein level, and its classification according to the number of modes.

## Supporting Information

S1 Supporting Information Derivation of stationary distributions. The supporting information includes mathematical derivations of steady state protein level distributions.
(PDF)

S1 Fig Robustness of the bimodal regime. The figure shows how Fig. 3 in the main text perturbs if the regulable burst rate *a*_1_ or mean burst size *b* are changed. As in Fig. 3, we assume that there are *y* = 100 binding sites which attract protein molecules with strong affinity (*k*_b_ = 1). In each row, the first panel shows a copy of Fig. 3 (*a*_1_ = 25, *b* = 50), while the other two show how that changes if *b* is varied (1st row), *a*_1_ is varied (2nd row), or both are varied in a manner that keeps maximal regulable production rate *a*_1_ *b* constant (3rd row). The specific choices of *a*_1_ and *b* are detailed above each panel.
(TIF)
