# Supplementary material for: Protein Copy Number Distributions for a Self-Regulating Gene in the Presence of Decoy Binding Sites
Source: PLoS One. 2015 Mar 26;10(3):e0120555. doi: 10.1371/journal.pone.0120555 (PMC4374843; doi:10.1371/journal.pone.0120555)
Supplement: S1 Supporting Information — The supporting information includes mathematical derivations of steady state protein level distributions. (PDF) [file pone.0120555.s001.pdf]

S1 Supporting Information: Derivation of stationary distributions

Pavol Bokes
Department of Applied Mathematics and Statistics
Comenius University, Bratislava, Slovakia
Email: pavol.bokes@fmph.uniba.sk

Abhyudai Singh
Department of Electrical and Computer Engineering
University of Delaware, Newark, Delaware, USA
Email: absingh@udel.edu

1 Master equation

The probability density function $p(x,t)$ of observing the protein at concentration x at time t satisfies the master equation

$$\frac{\partial p(x,t)}{\partial t} - \frac{\partial}{\partial x}(c(x)p(x,t)) = \int_0^x \left(b^{-1}\mathrm{e}^{-(x-x')/b} - \delta(x-x')\right) a(x')p(x',t)\mathrm{d}x', \tag{1}$$

where the dependences on the total protein level x of the decay rate $c(x)$ and burst rate $a(x)$ are given by

$$a(x) = a_0 + \frac{a_1 x_\mathrm{f}}{k_\mathrm{p} + x_\mathrm{f}}, \quad c(x) = \gamma_\mathrm{f} x_\mathrm{f} + \frac{\gamma_\mathrm{b} y x_\mathrm{f}}{x_\mathrm{f} + k_\mathrm{b}}, \tag{2}$$

in which

$$x_\mathrm{f} = \frac{x - y - k_\mathrm{b} + \sqrt{(k_\mathrm{b} + y - x)^2 + 4k_\mathrm{b}x}}{2} \tag{3}$$

gives the free protein level x_f as a function of the total protein level x, cf. Section 2 in the Main Text.

The stationary solution to (1) has been determined, using the Laplace transformation of the equation, for linear degradation rate (Friedman et al., 2006) and for a generic non-linear rate $c(x)$ (Mackey et al., 2013). For reader's convenience, we re-derive these results using a different method. Following that, the generic formula needs to be algebraically simplified to obtain a closed-form expression for the stationary distribution with our specific choices of $a(x)$ and $c(x)$ in mind.

Equation (1) can be written down in the form of

$$\frac{\partial p}{\partial t} + \frac{\partial J}{\partial x} = 0, \tag{4}$$

where the probability flux term is given by

$$J = -c(x)p(x,t) + \int_0^x \mathrm{e}^{-(x-x')/b} a(x')p(x',t)\mathrm{d}x'. \tag{5}$$

For the stationary solution $p(x,t) = p(x)$ both the flux J and its derivative $\mathrm{d}J/\mathrm{d}x$ must vanish, so that

$$c(x)p(x) = \int_0^x \mathrm{e}^{-(x-x')/b} a(x')p(x')\mathrm{d}x', \tag{6}$$

$$\frac{\mathrm{d}}{\mathrm{d}x}(c(x)p(x)) = a(x)p(x) - \frac{1}{b}\int_0^x \mathrm{e}^{-(x-x')/b} a(x')p(x')\mathrm{d}x'. \tag{7}$$

Dividing (6) by b and adding the resulting equation to (7) yields

$$\frac{\mathrm{d}}{\mathrm{d}x}(c(x)p(x)) + \frac{c(x)p(x)}{b} = a(x)p(x), \tag{8}$$

from which

$$p(x) = \frac{\kappa}{c(x)} \exp\left(-\frac{x}{b} + \int \frac{a(x)}{c(x)}\mathrm{d}x\right), \tag{9}$$

where κ is a normalisation constant; such a form has been disclosed in previous studies (Friedman et al., 2006; Mackey et al., 2013).

The convergence, as time t increases, of time-dependent solutions to the master equation (1) to the stationary distribution (9), in the L^1 sense, has been established in Mackey et al. (2013) under mild conditions on the functional form of $a(x)$ and $c(x)$. Specifically, Mackey et al. (2013) require

$$\int_0^\delta \frac{\mathrm{d}x}{c(x)} = \infty, \quad \int_0^\delta \frac{a(x)}{c(x)}\mathrm{d}x = \infty, \tag{10}$$

for a small $\delta > 0$, of which the former guarantees that the deterministic decay process does not lead to a (macroscopic) extinction, while the latter ensures that the waiting time for the next burst be finite. For our choices of $a(x)$ and $c(x)$, conditions (10) hold, since $a(x)$ is bound from below by $a_0 > 0$ and $c(x)$ is asymptotically linear for x small. In addition to (10), Mackey et al. (2013) require that the integral of (9) is finite and also that the mean decay rate $\int_0^\infty c(x)p(x)\mathrm{d}x$ be finite, both of which hold too for our choices of $a(x)$ and $c(x)$ due to asymptotic linearity of $c(x)$ for x very small or very large and also owing to boundedness of $a(x)$ from below as well as above, by positive constants.

2 Explicit steady-state solution

In this section we simplify the general formula (9) for stationary distribution, making use of the specific properties of our choices for the transcription and decay rates (2). We assume that time is measured in the units of free protein lifetime, i.e. that $\gamma_f = 1$.

By (2), we find that

$$\frac{a(x)}{c(x)} = \left(\frac{a_0}{x_\mathrm{f}} + \frac{a_1}{k_\mathrm{p} + x_\mathrm{f}}\right) \frac{x_\mathrm{f} + k_\mathrm{b}}{x_\mathrm{f} + k_\mathrm{b} + \gamma_\mathrm{b} y}. \tag{11}$$

Therefore, the integral in the exponential of (9) simplifies to

$$\int \frac{a(x)}{c(x)}\mathrm{d}x = \int \frac{a(x)}{c(x)}\frac{\mathrm{d}x}{\mathrm{d}x_\mathrm{f}}\mathrm{d}x_\mathrm{f} = \int \left(\frac{a_0}{x_\mathrm{f}} + \frac{a_1}{k_\mathrm{p}+x_\mathrm{f}}\right)\frac{x_\mathrm{f}+k_\mathrm{b}+\frac{yk_\mathrm{b}}{x_\mathrm{f}+k_\mathrm{b}}}{x_\mathrm{f}+k_\mathrm{b}+\gamma_\mathrm{b}y}\mathrm{d}x_\mathrm{f}$$
$$= q_1\ln x_\mathrm{f} + q_2\ln(x_\mathrm{f}+k_\mathrm{p}) + q_3\ln(x_\mathrm{f}+k_\mathrm{b}) + q_4\ln(x_\mathrm{f}+k_\mathrm{b}+\gamma_\mathrm{b}y), \tag{12}$$

where $\mathrm{d}x/\mathrm{d}x_\mathrm{f}$ is determined by differentiating

$$x = x_\mathrm{f} + \frac{yx_\mathrm{f}}{x_\mathrm{f}+k_\mathrm{b}}, \tag{13}$$

which expresses the total protein level x in terms of the free protein level x_f, cf. Section 2 in the Main Text. The integration of the rational fraction in (12) is done by partial fraction decomposition using software capable of symbolic calculation, and the coefficients multiplying the individual partial fractions are

$$q_1 = a_0\frac{k_\mathrm{b}+y}{k_\mathrm{b}+\gamma_\mathrm{b}y}, \tag{14}$$
$$q_2 = \frac{a_1(k_\mathrm{p}-k_\mathrm{b})}{k_\mathrm{p}-k_\mathrm{b}-\gamma_\mathrm{b}y}\left(1+\frac{yk_\mathrm{b}}{(k_\mathrm{p}-k_\mathrm{b})^2}\right), \tag{15}$$
$$q_3 = \frac{1}{\gamma_\mathrm{b}}\left(\frac{a_1k_\mathrm{b}}{k_\mathrm{p}-k_\mathrm{b}} - a_0\right), \tag{16}$$
$$q_4 = \left(\gamma_\mathrm{b}y + \frac{k_\mathrm{b}}{\gamma_\mathrm{b}}\right)\left(\frac{a_0}{k_\mathrm{b}+\gamma_\mathrm{b}y} - \frac{a_1}{k_\mathrm{p}-k_\mathrm{b}-\gamma_\mathrm{b}y}\right). \tag{17}$$

Substituting (12) into (9), we find that the total protein concentration x is given by the closed expression

$$p(x) = \kappa\exp\left(-\frac{x}{b}\right)x_\mathrm{f}^{q_1-1}(x_\mathrm{f}+k_\mathrm{p})^{q_2}(x_\mathrm{f}+k_\mathrm{b})^{q_3+1}(x_\mathrm{f}+k_\mathrm{b}+\gamma_\mathrm{b}y)^{q_4-1}, \tag{18}$$

in which x_f is understood to be a function of x, as given by expression (3). The formula (18) is valid as long as some special cases, namely that of $k_\mathrm{p} = k_\mathrm{b}$, or $k_\mathrm{p} = k_\mathrm{b} + \gamma_b y$, or $\gamma_b y = 0$, are avoided; should however any of these occur, the above integration procedure can easily be modified accordingly to obtain a valid result, as detailed below.

2.1 Special case $\gamma_\mathrm{b} = 0$

In this case, we have

$$\int \frac{a(x)}{c(x)}\mathrm{d}x = \int \frac{a(x)}{c(x)}\frac{\mathrm{d}x}{\mathrm{d}x_\mathrm{f}}\mathrm{d}x_\mathrm{f} = \int \left(\frac{a_0}{x_\mathrm{f}} + \frac{a_1}{k_\mathrm{p}+x_\mathrm{f}}\right)\left(1+\frac{yk_\mathrm{b}}{(x_\mathrm{f}+k_\mathrm{b})^2}\right)\mathrm{d}x_\mathrm{f}$$
$$= q_1\ln(x_\mathrm{f}) + q_2\ln(x_\mathrm{f}+k_\mathrm{p}) + q_3\ln(x_\mathrm{f}+k_\mathrm{b}) + q_4\frac{y}{x_\mathrm{f}+k_\mathrm{b}}, \tag{19}$$

where

$$q_1 = a_0 \left(1 + \frac{y}{k_\mathrm{b}}\right), \tag{20}$$

$$q_2 = a_1 \left(1 + \frac{yk_\mathrm{b}}{(k_\mathrm{b} - k_\mathrm{p})^2}\right), \tag{21}$$

$$q_3 = -\frac{y}{k_\mathrm{b}} \left(a_0 + \frac{a_1 k_\mathrm{b}^2}{(k_\mathrm{b} - k_\mathrm{p})^2}\right), \tag{22}$$

$$q_4 = a_0 - \frac{a_1 k_\mathrm{b}}{k_\mathrm{p} - k_\mathrm{b}}. \tag{23}$$

Substituting (19) into (9), we arrive at

$$p(x) = \kappa \mathrm{exp}\left(-\frac{x}{b} + q_4 \frac{y}{x_\mathrm{f} + k_\mathrm{b}}\right) x_\mathrm{f}^{q_1 - 1}(x_\mathrm{f} + k_\mathrm{p})^{q_2}(x_\mathrm{f} + k_\mathrm{b})^{q_3}, \tag{24}$$

where κ is the normalisation constant.

2.2 Special case $y = 0$

If $y = 0$, then (13) implies that $x = x_f$, and hence

$$\int \frac{a(x)}{c(x)} \mathrm{d}x = \int \frac{a_0}{x} + \frac{a_1}{x + k_\mathrm{p}} \mathrm{d}x = a_0 \mathrm{ln}(x) + a_1 \mathrm{ln}(x + k_\mathrm{p}). \tag{25}$$

Substituting (25) into (9), we find

$$p(x) = \kappa \mathrm{e}^{-x/b} x^{a_0 - 1}(x + k_\mathrm{p})^{a_1}, \tag{26}$$

where κ is the normalisation constant. This result can also be found in previous studies (Friedman et al., 2006; Mackey et al., 2013), which consider the model for burst-like gene expression in the absence of decoy binding sites.

2.3 Special case $k_\mathrm{p} = k_\mathrm{b}$

Here

$$\int \frac{a(x)}{c(x)} \mathrm{d}x = \int \frac{a(x)}{c(x)} \frac{\mathrm{d}x}{\mathrm{d}x_\mathrm{f}} \mathrm{d}x_\mathrm{f} = \int \left(\frac{a_0}{x_\mathrm{f}} + \frac{a_1}{k_\mathrm{b} + x_\mathrm{f}}\right) \frac{x_\mathrm{f} + k_\mathrm{b} + \frac{yk_\mathrm{b}}{x_\mathrm{f} + k_\mathrm{b}}}{x_\mathrm{f} + k_\mathrm{b} + \gamma_\mathrm{b} y} \mathrm{d}x_\mathrm{f}$$

$$= q_1 \mathrm{ln} x_\mathrm{f} + q_2 \mathrm{ln}(x_\mathrm{f} + k_\mathrm{b}) + q_3 \mathrm{ln}(x_\mathrm{f} + k_\mathrm{b} + \gamma_\mathrm{b} y) - \frac{a_1 k_\mathrm{b}}{\gamma_\mathrm{b}(k_\mathrm{b} + x_\mathrm{f})}, \tag{27}$$

where

$$q_1 = a_0 \frac{k_\mathrm{b} + y}{k_\mathrm{b} + \gamma_\mathrm{b} y}, \tag{28}$$

$$q_2 = -\frac{1}{\gamma_\mathrm{b}} \left(a_0 + \frac{a_1 k_\mathrm{b}}{\gamma_\mathrm{b} y}\right), \tag{29}$$

$$q_3 = \left(\gamma_\mathrm{b} y + \frac{k_\mathrm{b}}{\gamma_\mathrm{b}}\right) \left(\frac{a_1}{\gamma_\mathrm{b} y} + \frac{a_0}{k_\mathrm{b} + \gamma_\mathrm{b} y}\right). \tag{30}$$

Substituting (27) into (9), we find

$$p(x) = \kappa \mathrm{exp}\left(-\frac{x}{b} - \frac{a_1 k_\mathrm{b}}{\gamma_\mathrm{b}(k_\mathrm{b} + x_\mathrm{f})}\right) x_\mathrm{f}^{q_1 - 1}(x_\mathrm{f} + k_\mathrm{b})^{q_2 + 1}(x_\mathrm{f} + k_\mathrm{b} + \gamma_\mathrm{b} y)^{q_3 - 1}, \tag{31}$$

where κ is the normalisation constant.

2.4 Special case $k_\mathrm{p} = k_\mathrm{b} + \gamma_\mathrm{b} y$

Here

$$\int \frac{a(x)}{c(x)}\mathrm{d}x = \int \frac{a(x)}{c(x)}\frac{\mathrm{d}x}{\mathrm{d}x_\mathrm{f}}\mathrm{d}x_\mathrm{f} = \int \left(\frac{a_0}{x_\mathrm{f}} + \frac{a_1}{k_\mathrm{b} + \gamma_\mathrm{b} y + x_\mathrm{f}}\right) \frac{x_\mathrm{f} + k_\mathrm{b} + \frac{y k_\mathrm{b}}{x_\mathrm{f} + k_\mathrm{b}}}{x_\mathrm{f} + k_\mathrm{b} + \gamma_\mathrm{b} y}\mathrm{d}x_\mathrm{f}$$
$$= q_1 \mathrm{ln} x_\mathrm{f} + q_2 \mathrm{ln}(x_\mathrm{f} + k_\mathrm{b}) + q_3 \mathrm{ln}(x_\mathrm{f} + k_\mathrm{p}) + \frac{q_4}{k_\mathrm{b} + \gamma_\mathrm{b} y + x_\mathrm{f}}, \quad (32)$$

where

$$q_1 = \frac{a_0(k_\mathrm{b} + y)}{k_\mathrm{b} + \gamma_\mathrm{b} y}, \quad (33)$$
$$q_2 = \frac{1}{\gamma_\mathrm{b}}\left(\frac{a_1 k_\mathrm{b}}{\gamma_\mathrm{b} y} - a_0\right), \quad (34)$$
$$q_3 = a_1\left(1 - \frac{k_\mathrm{b}}{\gamma_\mathrm{b}^2 y}\right) + \frac{a_0 \gamma_\mathrm{b} y}{k_\mathrm{p}}\left(1 + \frac{k_\mathrm{b}}{\gamma_\mathrm{b}^2 y}\right), \quad (35)$$
$$q_4 = a_1\left(\gamma_\mathrm{b} y + \frac{k_\mathrm{b}}{\gamma_\mathrm{b}}\right). \quad (36)$$

Substituting (32) into (9), we find

$$p(x) = \kappa \exp\left(-\frac{x}{b} + \frac{q_4}{k_\mathrm{b} + \gamma_\mathrm{b} y + x_\mathrm{f}}\right) x_\mathrm{f}^{q_1 - 1}(x_\mathrm{f} + k_\mathrm{b})^{q_2 + 1}(x_\mathrm{f} + k_\mathrm{b} + \gamma_\mathrm{b} y)^{q_3 - 1}, \quad (37)$$

where κ is the normalisation constant.

2.5 Special case $k_\mathrm{p} = k_\mathrm{b}$ and $\gamma_\mathrm{b} = 0$

Here

$$\int \frac{a(x)}{c(x)}\mathrm{d}x = \int \frac{a(x)}{c(x)}\frac{\mathrm{d}x}{\mathrm{d}x_\mathrm{f}}\mathrm{d}x_\mathrm{f} = \int \left(\frac{a_0}{x_\mathrm{f}} + \frac{a_1}{x_\mathrm{f} + k_\mathrm{b}}\right)\left(1 + \frac{y k_\mathrm{b}}{(x_\mathrm{f} + k_\mathrm{b})^2}\right)\mathrm{d}x_\mathrm{f}$$
$$= q_1 \mathrm{ln}(x_\mathrm{f}) + q_2 \mathrm{ln}(x_\mathrm{f} + k_\mathrm{b}) + \frac{q_3}{x_\mathrm{f} + k_\mathrm{b}} + \frac{q_4}{(x_\mathrm{f} + k_\mathrm{b})^2}, \quad (38)$$

where

$$q_1 = a_0\left(1 + \frac{y}{k_\mathrm{b}}\right), \quad q_2 = a_1 - \frac{a_0 y}{k_\mathrm{b}}, \quad q_3 = a_0 y, \quad q_4 = -\frac{a_1 k_\mathrm{b} y}{2}. \quad (39)$$

Substituting (38) into (9), we find

$$p(x) = \kappa \mathrm{exp}\left(-\frac{x}{b} + \frac{q_3}{x_\mathrm{f} + k_\mathrm{b}} + \frac{q_4}{(x_\mathrm{f} + k_\mathrm{b})^2}\right) x_\mathrm{f}^{q_1 - 1}(x_\mathrm{f} + k_\mathrm{b})^{q_2}, \quad (40)$$

where κ is the normalisation constant.

References

N. Friedman, L. Cai, and X.S. Xie (2006). Linking stochastic dynamics to population distribution: an analytical framework of gene expression. *Phys. Rev. Lett.*, **97**, 168302.

M.C. Mackey, M. Tyran-Kaminska, and R. Yvinec (2013). Dynamic behavior of stochastic gene expression models in the presence of bursting. *SIAM J. Appl. Math.*, **73**, 1830–1852.
