# Supplementary figures and images for: Protein Copy Number Distributions for a Self-Regulating Gene in the Presence of Decoy Binding Sites
Source: PLoS One. 2015 Mar 26;10(3):e0120555. doi: 10.1371/journal.pone.0120555 (PMC4374843; doi:10.1371/journal.pone.0120555)

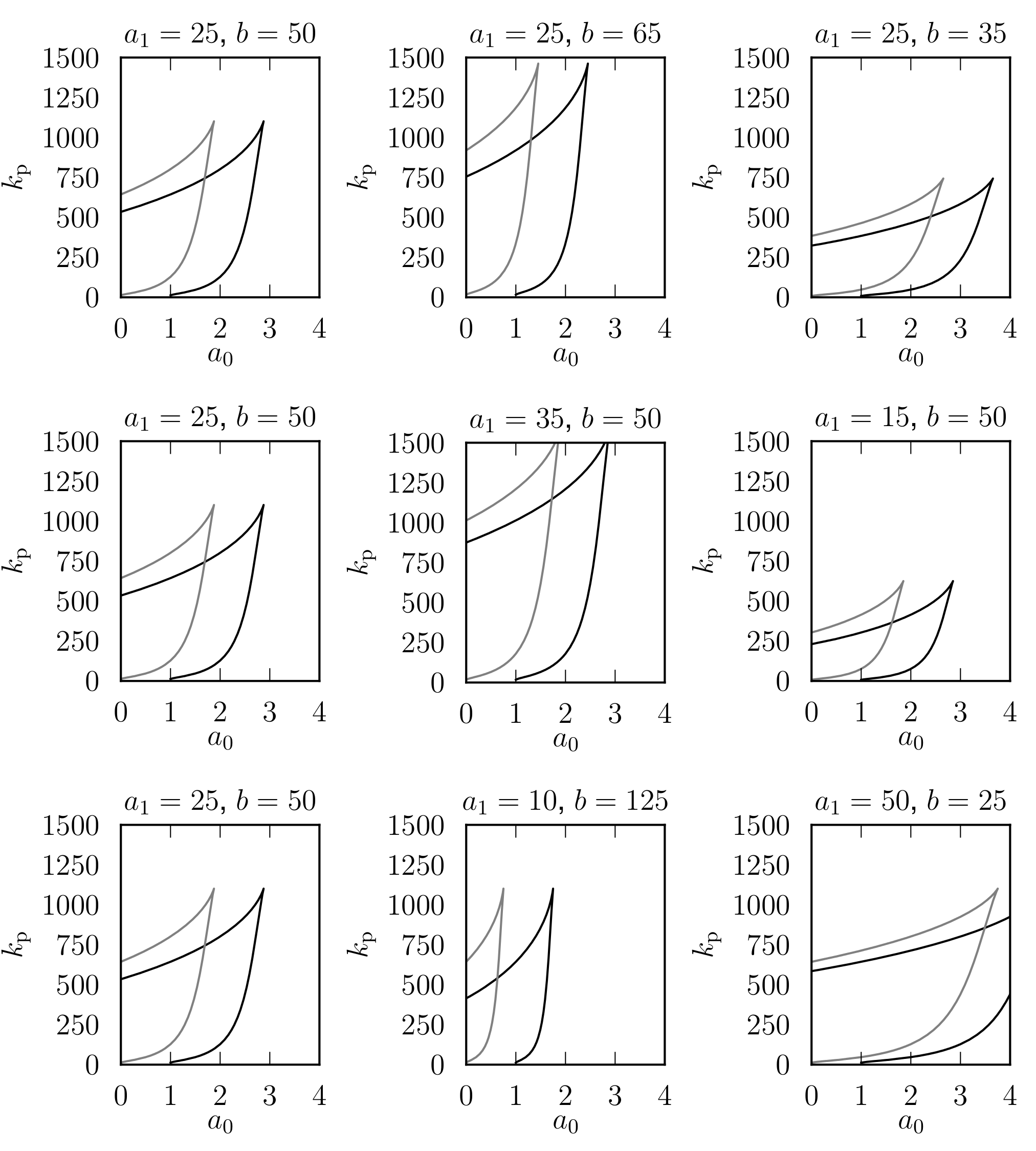

Supplement: S1 Fig — The figure shows how Fig. 3 in the main text perturbs if the regulable burst rate a 1 or mean burst size b are changed. As in Fig. 3, we assume that there are y = 100 binding sites which attract protein molecules with strong affinity (k b = 1). In each row, the first panel shows a copy of Fig. 3 (a 1 = 25, b = 50), while the other two show how that changes if b is varied (1st row), a 1 is varied (2nd row), or both are varied in a manner that keeps maximal regulable production rate a 1 b constant (3rd row). The specific choices of a 1 and b are detailed above each panel. (TIF) [file pone.0120555.s002.tif]
